# Legionnaires’ Disease Caused by *Legionella pneumophila* Serogroups 5 and 10, China

**DOI:** 10.3201/eid2007.131343

**Published:** 2014-07

**Authors:** Qi Zhang, Haijian Zhou, Rong Chen, Tian Qin, Hongyu Ren, Bin Liu, Xinliang Ding, Dan Sha, Weijie Zhou

**Affiliations:** Wuxi Center for Disease Control and Prevention, Wuxi, China (Q. Zhang, R. Chen, B. Liu, X. Ding, D. Sha, W. Zhou);; National Institute for Communicable Disease Control and Prevention, Beijing, China (H. Zhou, T. Qin, H. Ren);; State Key Laboratory for Infectious Disease Prevention and Control, Beijing (H. Zhou, T. Qin, H. Ren);; Collaborative Innovation Center for Diagnosis and Treatment of Infectious Diseases, Hangzhou, (H. Zhou, T. Qin)

**Keywords:** Legionnaires’ disease, Legionella pneumophila, bacteria, serogroup 5, serogroup 10, co-infection, pneumonia, China

**To the Editor:** Legionnaires’ disease is a systemic infection caused by gram-negative bacteria belonging to the genus *Legionella*. The primary clinical manifestation is pneumonia. *Legionella* spp. are typically found in natural and artificially hydrated environments.

*Legionella pneumophila* is the species responsible for ≈90% of human cases of infection. *L. pneumophila* is divided into 15 serogroups, among which serogroup 1 is the most prevalent disease-causing variant (*1*). In contrast, rare cases are caused by other serogroups. We describe a case of Legionnaires’ disease caused by co-infection with *L. pneumophila* serogroups 5 and 10 and the genotype characteristics of these strains.

The case-patient was a 77-year-old man who had chronic hepatitis B for 50 years, ankylosing spondylitis for 40 years, and chronic cholecystitis for 5 years. On September 17, 2012, he was admitted to Wuxi People’s Hospital (Wuxi, China) for treatment after a continuous cough for 15 days and a high fever for 2 days. At admission, the patient had a blood pressure of 130/65 mm Hg, a pulse rate of 102 beats/minute, and a body temperature of 37.4°C, which increased to 38.4°C four hours later. Laboratory tests showed a leukocyte count of 9,200 cells/μL (88.7% neutrophils) and a C-reactive protein level of 31 mg/L in serum. Lung inflammation was identified by computed tomography. The result of a urinary antigen test for *L. pneumophila* serogroup 1 (Binax, Portland, ME, USA) was negative. Bronchoalveolar lavage was performed, and fluid was collected for bacterial culture and molecular analysis.

Real-time PCRs were performed with primers specific for the 5S rRNA gene of the genus *Legionella* (*2*) and the *L. pneumophila*-specific *mip* gene (*3*). *Legionella* colonies isolated from bronchoalveolar lavage fluid grew on buffered-charcoal yeast extract agar. Nine *Legionella*-like colonies were isolated, and all showed positive results by PCRs. The colonies were identified as *L. pneumophila* serogroups 2–14 by using the *Legionella* latex test (Oxoid, Basingstoke, UK). Among these colonies, 5 were identified as *L. pneumophila* serogroup 5, and 4 were identified as serogroup 10 by using a monoclonal antibody (Denka Seiken, Tokyo, Japan). Environmental investigations were conducted in the patient’s house and hospital room, but *L. pneumophila* serogroup 5 and 10 were not detected in any of the locations tested.

Pulsed-field gel electrophoresis (PFGE) (*4*) was used to investigate the 9 *L. pneumophila* strains. Two PFGE patterns that were 94% similar were observed; each pattern represented 1 serogroup. The PFGE patterns were compared with those of a reference database of *L. pneumophila* for China. All *L. pneumophila* in the database, including 41 strains isolated from the city in which the patient resided in 2012, had patterns different from those of the 9 strains.

Two clinical *L. pneumophila* strains of different serogroups were further analyzed by sequence typing (*5*,*6*). Sequence type (ST) indicated that allele numbers for *flaA*, *pilE*, *asd*, *mip*, *mompS*, *proA*, and *neuA* genes were 6, 10, 15, 28, 21, 7, and 207 for serogroup 5 strains and 6, 10, 15, 10, 21, 40, and 207 for serogroup 10 strains. By querying the ST database for *L*. *pneumophila* (http://www.ewgli.org), we found that both profiles were new and assigned these 2 strains the numbers ST1440 (serogroup 5) and ST1439 (serogroup 10). STs of these 2 isolates differed from each other by only 2 alleles (3 nt in the *mip* gene and 1 nt in the *proA* gene), which suggested that the isolates might be more closely related to each other than suggested by serologic analysis.

Human infections with *L. pneumophila* serogroups 5 and 10 have been rarely reported (*1*,*7*). Our study confirms human infection with 2 *L. pneumophila* serogroups that did not involve serogroup 1. Results for this case-patient also indicated that a negative urinary antigen test result should not be a reason for ruling out Legionnaires’ disease because the urinary antigen kit used detects only *L. pneumophila* serogroup 1 antigen. *L. pneumophila* serogroups 5 and 10 are probably underrecognized pathogenic serogroups. Culture and molecular analysis should be performed to obtain an accurate diagnosis. Rare co-infections with *L. pneumophila* serogroup strains have been identified by culture methods (*8*,*9*).

The cases reported previously and in this study indicate that co-infections with different serogroups are more common than currently recognized and that multiple colonies should be tested for accurate epidemiologic investigations. Qin et al. reported that pathogenic *Legionella* strains of different species, serogroups, and genotypes were isolated from the same hot spring water samples (*10*). This finding suggests that co-infections with different *Legionella* strains may occur under certain conditions.

In China, Legionnaires’ disease is usually ignored in the differential diagnosis of pneumonia because most clinicians lack experience with this disease. This case highlights the need to familiarize physicians with diagnostic methods for identifying *Legionella* pneumonia in clinics in China and for further epidemiologic surveillance to monitor this disease and improve public health disease control strategies.
